# Rehabilitation Nursing Care for Older Adults with Impaired Fine Motor Function: From Design to Validation

**DOI:** 10.3390/nursrep16010008

**Published:** 2025-12-24

**Authors:** Magda Rafaela Carneiro Freitas, Ana da Conceição Alves Faria, Carla Gomes da Rocha, Maria Narcisa da Costa Gonçalves, Olga Maria Pimenta Lopes Ribeiro

**Affiliations:** 1 Integrated Continuing Care Unit of Medium Duration and Rehabilitation of S. Pedro, 4810-178 Guimarães, Portugal; 806915@unilabs.com; 2 Department of Nursing, Santa Maria Health School, 4049-024 Porto, Portugal; ana.faria@santamariasaude.pt; 3 Department of Nursing, School of Health Sciences, HES-SO Valais-Wallis, 1950 Sion, Switzerland; carla.gomesdarocha@hevs.ch; 4 Department of Nursing, Nursing School of Porto, 4200-072 Porto, Portugal; 5 RISE-Health, 4200-319 Porto, Portugal

**Keywords:** rehabilitation nursing, fine motor function, aged, old age assistance, stroke rehabilitation

## Abstract

**Background:** Population ageing and the growing prevalence of chronic diseases, particularly stroke, have negative repercussions on fine motor function, compromising the independence of older adults. The Specialist Nurse in Rehabilitation Nursing plays a central role in functional recovery and in improving quality of life. This study aims to describe the process of developing and validating the design of rehabilitation nursing care for older adults with impaired fine motor function. **Methods:** This paper is a three-phase methodological study conducted between January and July 2025: (1) initial development of the design of rehabilitation nursing care for older adults with impaired fine motor function; (2) validation of the content of the proposed design, using the modified e-Delphi technique; and (3) development of the final model of the care design. **Results:** The e-Delphi study, involving a panel of 15 experts, allowed the content validation of the design of rehabilitation nursing care for older adults with impaired fine motor function after two rounds. Following the suggestions, the final care design model, in relation to fine motor function, comprises five steps: (1) collection of relevant data, (2) identification of possible nursing diagnoses, (3) definition of objectives, (4) planning and implementation of interventions, and (5) evaluation of outcomes. As part of step 4, photographic records of exercises focused on the recovery of fine motor function were included. **Conclusions:** The final model of the design of rehabilitation nursing care for older adults with impaired fine motor function, developed and validated in this study, may serve as a guiding framework in the delivery of specialised care to this population.

## 1. Introduction

The increase in life expectancy and the consequent demographic ageing, both nationally and internationally, have led to a growing number of older adults with health problems and in situations of dependency [1]. At the European level, recent demographic projections from the European Commission indicate a sustained ageing trend, marked by a growing proportion of older adults and a shrinking working-age population, placing additional demands on health and social care systems. In Portugal, it is estimated that by 2050, the older population will represent around 32% of the total population [2]. Ageing constitutes a critical stage of the life cycle, and from a biopsychosocial perspective, it is imperative to consider its specificities, particularly the needs, challenges, and health conditions associated with it [3].

Although ageing is not classified as a disease, it is estimated that more than 80% of older adults present with one or more pathological processes, with repercussions at the physical, psychological, social, and economic levels [4,5]. Among these conditions, stroke stands out due to its high prevalence [4]. It is projected that by 2047, the number of people living with stroke sequelae will increase by 27%, a fact explained by the projected demographic changes, which point to the continuation of population ageing [6].

Stroke represents the second leading cause of death and the main cause of disability in adults [7,8]. The subsequent clinical manifestations, resulting from ischaemic or haemorrhagic events, are characterised by considerable heterogeneity. The resulting disability, of a multifactorial nature, is determined by the location of the brain lesion, the individual’s previous functional condition, and the degree of neurological recovery achieved [9,10].

The approach to the older person with nervous system impairment resulting from stroke is characterised by its breadth and complexity, involving multiple areas that require the attention of health professionals, particularly Specialist Nurses in Rehabilitation Nursing (SNRNs). In Portugal, SNRN are advanced practice nurses formally certified by the Order of Nurses, with specialised and autonomous additional skills in the assessment, planning, and implementation of rehabilitation-focused interventions. Internationally, these professionals stand out for their essential contribution to improving functional outcomes, promoting autonomy, preventing disability, and strengthening the quality, safety, and efficiency of care delivery. These include muscle strength, muscle tone, joint movement, fine motor function, balance, somatic sensations, swallowing, and communication [11].

Investment in all these areas of care is pertinent; however, given the consequences of ageing and stroke, the domain of fine motor function assumes particular relevance. Indeed, even in the presence of significant neurological recovery, the absence of specific and early intervention in this domain may markedly compromise the autonomy and quality of life of the older person [12,13]. According to Meleis’ Transition Theory, fine motor impairment in older adults represents a transitional experience that affects functional capacity, identity, autonomy and adaptation. Integrating this theoretical perspective strengthens the conceptual foundation of the proposed care design by emphasising the relationship between motor recovery, adaptive processes and the person’s engagement in self-care.

In the International Classification of Functioning, Disability and Health (ICF), fine movements refer to the performance of coordinated actions to handle, lift, manipulate, and release objects using the hands, fingers, and thumb—for example, picking up coins from a table or turning a knob or door handle [14].

In the International Classification for Nursing Practice (ICNP^®^), fine motor function is defined as the spontaneous, voluntary, or involuntary movement of muscles and joints [15].

From a more specific perspective, fine motor function can be defined as the ability to control small muscle groups in order to perform fine skills, requiring concentration, movement organisation, and visuomotor coordination. This function depends on precise movements of the hands and fingers, which demand complex cerebral coordination. Such movements are considered sophisticated control, as they involve the multidimensional engagement of dexterity, coordination, and stability of the hands and fingers [16,17].

Indeed, fine motor function results from the interaction between multiple sensory and motor systems and is essential for holding, grasping, and manipulating objects [18]. This competence assumes particular relevance in movements associated with self-care, since these often require the coordinated manipulation of objects with both hands. The performance of the pincer movement, in particular, depends on the integration of motor coordination, proprioception, and joint integrity [19].

A study conducted by Herring-Marler et al. (2014) concluded that deficits in fine motor function are significant and, even when exclusively associated with the ageing process, may cause severe limitations in activities of daily living (ADL) [20]. When, in addition to the developmental transition inherent to ageing, a health/illness transition also occurs, fine motor function impairment tends to worsen [11,21]. One of the main sequelae after stroke is the inability to perform independent finger movements, with around two-thirds of people who have experienced a stroke presenting with functional deficits in the upper limbs, particularly in the hand [22].

In addition, Johansson and Häger (2019) highlight that limitations in upper limb dexterity persist in 45% to 70% of patients one year after the cerebrovascular event [23]. Indeed, the majority of people who have had a stroke present with long-term persistent upper limb dysfunction. Only between 15% and 33% of cases show functional recovery, while in approximately 66% no improvement is observed within the first six months after the event [24].

Thus, with the aim of intervening and promoting an improvement in the person’s quality of life, the functional re-education of the hand assumes particular relevance, as it is directly associated with the main cause of loss of skills, with immediate repercussions on activities of daily living [25,26,27]. Functional rehabilitation of the hand is based on activities designed to prevent dysfunction, restore functionality, and avoid progression to the point of compromising the person’s ability to effectively perform their daily tasks [28].

In the specific context of rehabilitation, it is recommended that the person be encouraged to carry out small household tasks, such as holding light utensils, as well as to participate in self-care activities, namely dressing and undressing, grooming, and ensuring personal hygiene. These practices promote mobilisation of the hand, facilitate body integration, and contribute to reducing the perception of disability and uselessness often associated with functional impairment [24,25,28].

Regardless of the context in which they practise, the SNRN frequently works with older adults who, as a result of the ageing process and/or a pathological process—particularly stroke—present with care needs across multiple domains [11]. This professional plays a central role in the early diagnosis of the health needs of older adults, as well as in the design and implementation of plans and programmes that maximise their functional capacities, prevent complications associated with ageing or pathological processes, and promote inclusion and social participation [29].

Although investment in all domains of bodily and adaptive processes is essential, in the acute phase, during hospitalisation, the design and delivery of care are often constrained by the high care needs presented by older adults and by the resources available, which frequently imposes the need to define priority areas of intervention [30]. Within this prioritisation, interventions aimed at improving fine motor function tend to be relegated to a secondary level, further compromising the ability to perform activities inherent to self-care [30]. Despite the existence of regulations [29,31], the systematisation of SNRN interventions directed towards older adults with impaired fine motor function remains scarce. Although established rehabilitation frameworks such as the International Classification of Functioning, Disability and Health (ICF) provide a comprehensive conceptual model for understanding functioning and disability, they are insufficient to address the specific and growing needs of this population. The ICF does not offer operational guidance for the systematic assessment, planning and implementation of nursing-led fine motor rehabilitation interventions. Furthermore, existing guidelines are often generic, fragmented or focused primarily on global motor rehabilitation, lacking specificity for nursing practice and failing to integrate the multidimensional nature of ageing, functional decline and dependency. The proposed model aims to address these gaps by providing a structured, evidence-based and nursing-centred framework that organises care into clearly defined stages, supports clinical decision-making, and promotes consistency and quality in rehabilitation nursing interventions for this population.

In view of the above and the need to invest in this area, and within the scope of a broader project, this study aims to describe the process of developing and validating the design of rehabilitation nursing care for older adults with impaired fine motor function.

## 2. Materials and Methods

This study followed a three-phase methodological design. It is important to note that this is not a clinical trial involving patients, but rather a content validation study through expert panel consensus [32]. In this regard, this study was conducted in three distinct phases: (1) initial development of the design of rehabilitation nursing care for older adults with impaired fine motor function; (2) content validation of the proposed design, using the modified e-Delphi technique; and (3) preparation of the final model of the design of rehabilitation nursing care for older adults with impaired fine motor function, based on the contributions obtained in the previous phases.

### 2.1. Study Design

In Phase I, the design of rehabilitation nursing care for older adults with impaired fine motor function was developed. This initial phase was informed by the literature review [30], the regulatory instruments governing the professional practice of SNRNs in Portugal [29,31,33], and the professional experience of the authors, all specialists in rehabilitation nursing. A structured narrative synthesis was conducted using peer-reviewed publications, international rehabilitation frameworks (e.g., ICF), national regulatory standards, and clinical guidelines. All authors independently reviewed the materials, extracted key concepts, and compared interpretations to ensure consistency, resolving divergences through discussion until consensus was reached.

For the development of the care design, it was taken into account that responding to people’s problems and needs requires a systematic and intentional approach, the implementation of which depends on the use of a scientific methodology [11]. Thus, the care design in the domain of fine motor function included: (1) identification of relevant data; (2) definition of possible diagnoses; (3) identification of objectives; (4) planning and implementation of interventions; and (5) evaluation of outcomes achieved. Within stage 1—collection of relevant data—it was considered that there are instruments which may be highly useful in the process of clinical decision-making when caring for a person with impaired fine motor function. Among these instruments, the Nine Hole Peg Test stands out. In this test, the individual is asked to remove nine pegs from a container and insert them into a board, one at a time. They must then be removed as quickly as possible and placed back into the container. The order of insertion and removal of the pegs is not specified, and there are more than 360,000 possible ways to insert the pegs. It consists of a measure for assessing manual dexterity in people with conditions affecting fine motor function. The person’s performance is scored according to the speed with which they are able to insert and remove the pegs: the faster the performance, the better the result. If a peg falls off the board, the test is interrupted and restarted. The results are compared with normative values for the general population, thereby determining whether the performance is within the expected range or indicates impairment. Furthermore, it allows the observation of movement quality, coordination, and the presence of tremors or other signs of motor difficulty [12,23,34].

In stages 2—definition of possible diagnoses, 3—identification of objectives, 4—planning and implementation of interventions, and 5—evaluation of outcomes—the contributions of the International Classification for Nursing Practice [15] and of the Nursing Ontology [35] were taken into consideration. It is important to note that within stage 4—planning and implementation of interventions—for the systematisation of fine motor function exercises, the contributions of several authors were considered [36,37,38]. Once this phase was completed, it became pertinent to validate the content included in the care design through expert consensus in the field, with the aim of aligning the stages with the reality of rehabilitation nursing practice directed towards older adults with impaired fine motor function.

Thus, in Phase II, a modified e-Delphi study was conducted to validate the content integrated into the care design. Consensus among the experts was achieved after two rounds. To ensure rigour, this study was conducted in accordance with the Conducting and Reporting of Delphi Studies (CREDES) recommendations [39].

In Phase III, following validation of the content integrated into the care design, the final model of the rehabilitation nursing care design for older adults with impaired fine motor function was developed. Considering that one of the main objectives is to promote systematisation in the domain of fine motor function, and in order to facilitate understanding and follow the experts’ suggestions, photographic records of some interventions were produced.

### 2.2. Participants

In Phase II, the selection of experts was carried out through non-probabilistic, purposive sampling. In this study, “experts” were considered to be professionals with in-depth knowledge and consolidated experience in the field of rehabilitation nursing care directed towards older adults with impaired fine motor function. The inclusion criteria were: (a) being a SNRN, regardless of whether their employer formally recognised their specialist status in the employment contract—a distinction that reflects specificities of the Portuguese regulatory framework, in which the professional title may not always align with the institutional job title; (b) having experience, knowledge, or involvement in research projects in the field of rehabilitation nursing care directed towards older adults; and (c) demonstrating availability and willingness to participate in all rounds of the e-Delphi. A total of 17 experts were selected. To enhance methodological transparency, the study followed the Conducting and Reporting of Delphi Studies (CREDES) guidance. Expert recruitment used purposive sampling to ensure representation across different practice settings (acute care, rehabilitation units, long-term care and community services) and geographical areas in Portugal. The sample size (17 invited; 15 respondents) followed international recommendations indicating that panels of 10–18 experts are adequate for content validation studies in specialised clinical fields.

Contact with the participants was made by email, which included a formal invitation letter and a link to access the study. Upon accessing the study, participants were informed about the objectives, rationale, potential benefits, and ethical aspects of the study, including informed consent, which they had to accept before beginning the questionnaire. Confidentiality was ensured, and all participants were duly informed that they could withdraw from the study at any time without any consequences.

### 2.3. Data Collection and Analysis Procedures

In Phase II, when conducting the modified e-Delphi, it was assumed that consensus obtained from a group of experts is more robust and reliable than isolated individual opinions. The content of the rehabilitation nursing care design for older adults with impaired fine motor function was evaluated by the experts using a four-point Likert scale: (1) strongly disagree; (2) partially disagree; (3) agree; and (4) strongly agree.

The data obtained from the experts’ validation were organised and subsequently analysed using the Content Validity Index (CVI) [32]. The CVI was calculated by dividing the number of responses scored as 3 or 4 by the total number of responses. Based on the results, items with a CVI below 0.90 were reviewed and/or reformulated, with a CVI equal to or greater than 0.90 being adopted as the validity criterion [32].

#### 2.3.1. First Round

The questionnaire was developed using the Microsoft Forms platform and structured in two sections: the first dedicated to the participants’ sociodemographic and professional characteristics; and the second to the presentation of the content of the rehabilitation nursing care design for older adults with impaired fine motor function. For items evaluated as “strongly disagree” or “partially disagree”, the experts were invited to suggest improvements. At the end of the questionnaire, an open-ended space was also provided for additional suggestions from the experts.

It is important to note that the questionnaire was pre-tested with three nurses who met the inclusion criteria but were not included in the study sample, which allowed assessment of the clarity and comprehensibility of the questions.

Based on the CVI calculations and the experts’ suggestions, the content of the care design was analysed individually, stage by stage, enabling the necessary changes to be made.

#### 2.3.2. Second Round

After incorporating the feedback obtained in the first round, a revised version of the rehabilitation nursing care design for older adults with impaired fine motor function was sent to the experts in the second round. All validation steps were repeated as in the first round, allowing the stability of the responses to be assessed. As before, at the end of the questionnaire, all experts were invited to provide additional suggestions. The scores assigned and qualitative suggestions were analysed to finalise content validation.

Descriptive statistics were used to analyse the sociodemographic and professional characteristics of the participants and to calculate the CVI.

Response stability, which determines whether additional rounds are needed, was assessed by comparing item-level CVI values between rounds, examining the consistency of experts’ ratings and verifying the absence of new substantive suggestions in Round 2. Qualitative comments provided in both rounds were subjected to thematic categorisation and used to refine wording, clarify concepts or adjust the organisation of items within the care design. Strategies to minimise attrition included reminder emails and flexible completion timelines.

### 2.4. Ethical Considerations

The development and validation of the rehabilitation nursing care design for older adults with impaired fine motor function was integrated into a broader research project, which received approval from the Ethics Committee (reference no. 024/2020). It is important to note that all participants provided informed consent, and that all data collected were duly coded and treated confidentially.

## 3. Results

The results are presented based on the three phases: (1) initial development of the design of rehabilitation nursing care for older adults with impaired fine motor function; (2) content validation of the proposed care design, using the modified e-Delphi technique; (3) development of the final model of the design of rehabilitation nursing care for older adults with impaired fine motor function.

### 3.1. Phase I—Initial Development of the Design of Rehabilitation Nursing Care for Older Adults with Impaired Fine Motor Function

In the first phase of the study, the design of rehabilitation nursing care for older adults with impaired fine motor function was developed. In line with the stages of the nursing process, the care design was structured into five steps: (1) collection of relevant data; (2) identification of possible nursing diagnoses; (3) definition of objectives; (4) planning and implementation of interventions; and (5) evaluation of outcomes.

During the development of the initial proposal, within step 1—collection of relevant data—in addition to the assessment of the person’s clinical condition, supporting instruments for the process of clinical decision-making in people with impaired fine motor function were considered.

With regard to steps 2 and 3—identification of possible nursing diagnoses and definition of objectives—the needs and problems emerging from function, as well as from the adaptive process related to developmental transition (within the scope of ageing) and/or health/illness transition (within the scope of stroke), experienced by the person were taken into account.

In step 4—planning and implementation of interventions—the systematisation of fine motor function exercises was carried out. The final step—evaluation of outcomes—corresponds to the moment when the SNRN analyses whether the objectives previously defined were fully or partially achieved, as a result of the interventions implemented. This evaluation not only verifies whether changes have occurred in the status of the diagnoses but also involves a critical and reflective analysis that allows for confirming improvements, identifying persistent needs, and reorienting the care plan in a dynamic and individualised manner.

### 3.2. Phase II—Validation of the Content of the Proposed Care Design

In the first round of the modified e-Delphi experiment, 17 experts were contacted and invited, of whom 15 agreed to participate and completed the questionnaire, achieving a response rate of 90%. In the second round, all experts responded within the defined timeframe. Table 1 presents the sociodemographic and professional characteristics of the experts who participated in both rounds of the modified e-Delphi experiment.

In the first round, regarding the question “does the care design meet the needs of older adults with impaired fine motor function?”, 100.0% agreement was obtained.

With respect to the content of each step, the CVI was calculated. Based on the content validation, the steps that individually obtained a CVI below 0.90 were reviewed and adjusted. Thus, in light of the improvement suggestions provided by the experts, two steps were adjusted: identification of possible nursing diagnoses, and planning and implementation of interventions. After incorporating the suggested improvements, the second round was carried out. The changes made to the two steps resulted in an improvement of the CVI, which exceeded 0.90 in all steps (Table 2).

Items related to nursing diagnoses and intervention planning generated the greatest divergence in Round 1, based mainly on requests for clarity or alignment with clinical practice. These items were revised linguistically and conceptually, leading to full consensus in Round 2.

Following the improvement suggestions regarding the step “identification of possible nursing diagnoses”, in addition to potential to improve capacity, the diagnosis “potential to improve knowledge about fine motor function exercises” was added. With respect to the step “planning and implementation of interventions”, in addition to the description of the exercises, photographic records of the exercises were included.

### 3.3. Development of the Final Model of the Rehabilitation Nursing Care Design for Older Adults with Impaired Fine Motor Function

Following the proposed rehabilitation nursing care design for older adults with impaired fine motor function, the final model comprises the five steps already mentioned: (1) collection of relevant data; (2) identification of possible nursing diagnoses; (3) definition of objectives; (4) planning and implementation of interventions; and (5) evaluation of outcomes.

#### 3.3.1. Collection of Relevant Data

With regard to the person’s clinical condition, the relevant data point to difficulties in handling small objects, increased execution time in the Nine Hole Peg Test, and reduced quality of movements associated with activities of daily living.

Concerning the adaptive process, the following are considered relevant data: lack of awareness of the relationship between exercises and fine motor function; lack of knowledge about fine motor function exercises; lack of ability to perform fine motor function exercises; and the perception that training in fine motor function is burdensome.

Cognitive capacity, physical capacity, awareness of changes in health status, willpower, engagement in the learning process, demonstrated belief in the ability to recover, and the expressed desire to become more independent are all conditions that should be assessed, as they may facilitate or hinder the clinical progression of the older person and therefore constitute relevant data.

#### 3.3.2. Identification of Possible Nursing Diagnoses

Based on the data collected, the possible nursing diagnoses are (1) impaired fine motor function, (2) potential to improve awareness of the association between exercises and fine motor function, (3) potential to improve knowledge about fine motor function exercises, (4) potential to improve ability to perform fine motor function exercises, and (5) potential to improve the meaning attributed to fine motor function training.

#### 3.3.3. Definition of Objectives

Following the nursing diagnosis related to the clinical condition—impaired fine motor function—the main objective is to determine the progression of fine motor function. With regard to the nursing diagnoses within the adaptive process, the main objective is to promote the person’s adherence to fine motor function training, which consequently increases the potential to improve fine motor function.

#### 3.3.4. Planning and Implementation of Interventions

Following the identified diagnoses and the objectives defined, specific interventions emerged. In relation to the diagnosis “impaired fine motor function”, the intervention “Assess the progression of fine motor function” is highlighted.

For the diagnosis “potential to improve awareness of the relationship between exercises and fine motor function”, the interventions “Assess progression of awareness of the relationship between exercises and fine motor function” and “Provide experience to promote awareness of the relationship between exercises and fine motor function” emerged.

With respect to the diagnosis “potential to improve knowledge about fine motor function exercises”, the interventions “Assess progression of knowledge about fine motor function exercises” and “Teach about fine motor function exercises” are emphasised.

With regard to the diagnosis “potential to improve the ability to perform fine motor function exercises”, the following interventions are emphasised: “Assess progression of the ability to perform fine motor function exercises”, “Instruct on fine motor function exercises”, “Train fine motor function”, and “Assess progression of adherence to fine motor function training”.

For the diagnosis “potential to improve the meaning attributed to fine motor function training”, the interventions “Assess progression of the meaning attributed to fine motor function training” and “Assist the person in analysing the hindering meaning” are highlighted.

Given the impossibility of specifying all interventions in this article, it was decided to detail the interventions “Assess the progression of fine motor function” and “Instruct on fine motor function exercises”.

**Figure 1:**
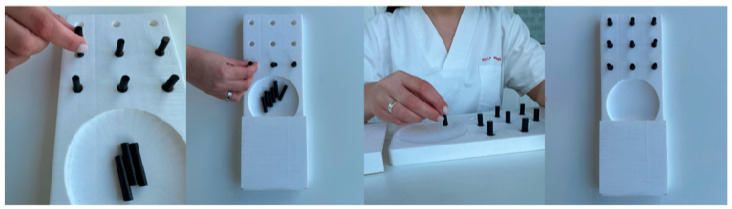
Representation of the performance of the Nine Hole Peg Test. Figure created by the authors.

To assess the progression of fine motor function, a tool specifically adopted by the authors for this purpose—the Nine Hole Peg Test (Figure 1)—will be used. For this intervention, the person should be comfortably seated with their back properly supported and their arms resting on a table, keeping the elbows flexed at 90° and the forearms in pronation. The task begins with the removal of the pegs placed in the board. These should be removed one at a time and inserted into the holes, with no specified order of insertion. After all the pegs have been inserted, the person should then remove them, also one at a time, and place them back into the board, again without a predetermined order. Throughout the procedure, the person should be instructed to perform the task as quickly as possible. The time required to complete the test is recorded using a stopwatch. Whenever a peg falls outside the board, the test should be interrupted and restarted.

With regard to the intervention “Instruct on fine motor function exercises”, the exercises and resources used for their performance are presented below.

**Figure 2:**
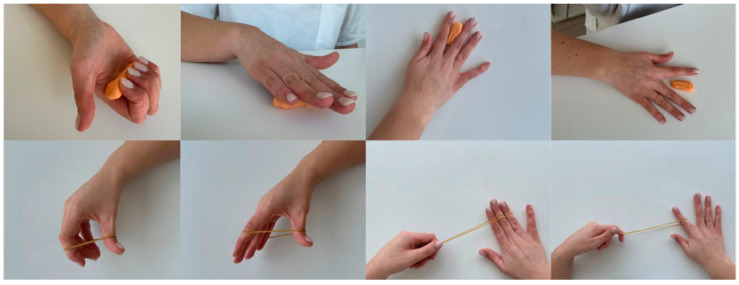
Representation of flexion, extension, adduction, and abduction finger exercises using modelling clay and elastic bands. Figure created by the authors.

For the execution of flexion, extension, adduction, and abduction movements of the fingers, resources such as modelling clay and elastic bands may be used, with the SNRN demonstrating the execution of each movement beforehand. It is recommended that each exercise be repeated ten times for each finger. Figure 2 illustrates the exercises described.

**Figure 3:**
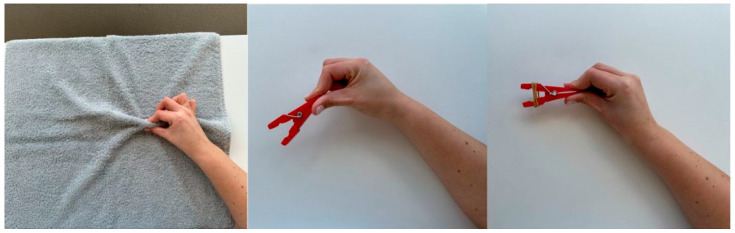
Representation of the progression of complexity levels in pincer movement exercises. Figure created by the authors.

For the performance of pincer movement exercises, the use of different resources is recommended in order to progressively increase the complexity of the task. At the simplest level, a towel may be used, demonstrating to the person how to wrinkle it with the fingers in a pincer movement, initially using all fingers together and then alternating the use of each finger. At the intermediate level, a peg is used: the person should be instructed to perform the pincer movement, first with the 1st and 2nd fingers of the hand, and then alternating the use of the remaining fingers, in order to grasp the peg fixed to a surface, open it, and place it on another surface. At the most complex level, an adapted peg with an elastic band at the end is used, demonstrating to the person how to perform the pincer movement while alternating the use of each finger of the hand. It is recommended that each exercise be repeated ten times per finger. Figure 3 presents the exercises described.

**Figure 4:**
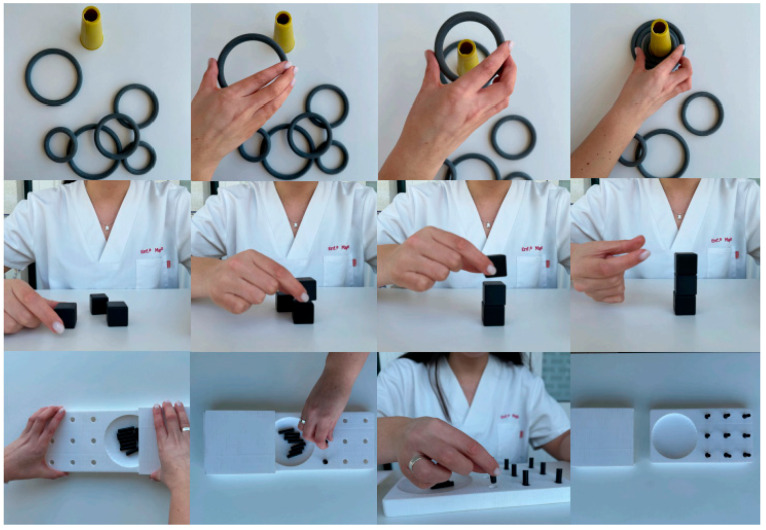
Representation of the progression of complexity levels in fine motor function exercises using rings, cubes, and pegs. Figure created by the authors.

Following the exercises aimed at promoting fine motor function, the use of different resources makes it possible to structure the progression of the task, gradually increasing its level of complexity. At the simplest level, rings may be used: the person should be guided to pick up each one individually, place it on a cone, and then remove them one by one with the hand. To increase difficulty, rings of progressively smaller sizes may be provided. At the intermediate level, 3 × 3 cm cubes are used: the person should be guided to stack them successively and then remove them one by one. At the most complex level, pegs are used: the person should be instructed to pick up each peg individually, insert it into a board with holes, and then remove them one by one. It is recommended that each exercise be repeated ten times with each finger. Figure 4 presents the exercises described.

**Figure 5:**
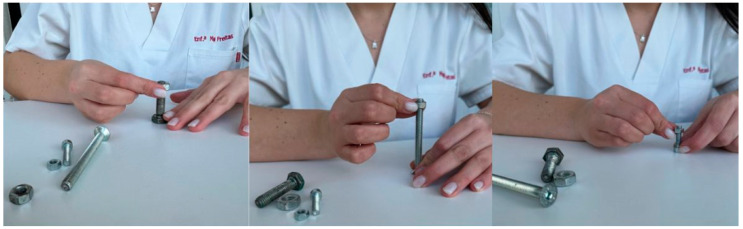
Representation of fine motor function exercises using bolts and nuts. Figure created by the authors.

In addition to the exercises already presented, bolts and nuts may be used to improve fine motor function (demonstrating to the person how to screw the nut onto the bolt, initially with the 1st and 2nd fingers of the hand and subsequently alternating the use of the remaining fingers). To increase the difficulty of the exercise, bolts and nuts of progressively smaller sizes should be provided. It is recommended that each exercise be repeated ten times per finger. Figure 5 presents the exercise described.

**Figure 6:**
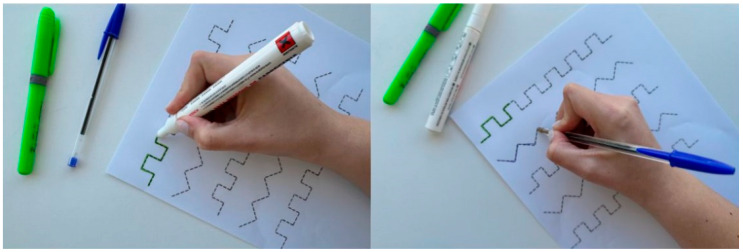
Representation of writing exercises using markers and a pen. Figure created by the authors.

In addition to the exercises previously described, fine motor function can also be improved through exercises using markers, initially demonstrating to the person the execution of writing activities, such as tracing pre-drawn paths on sheets prepared for this purpose. To increase the complexity of the exercise, the use of pens is recommended. Figure 6 illustrates the exercise described.

**Figure 7:**
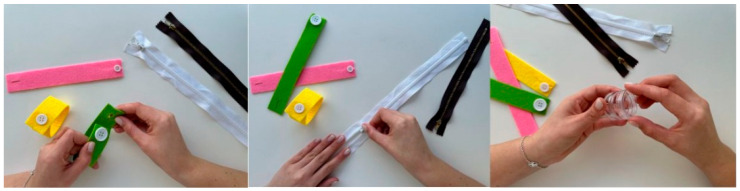
Representation of activities of daily living exercises. Figure created by the authors.

Additionally, to improve fine motor function, exercises involving the manipulation of objects associated with activities of daily living may also be performed, such as opening zips, handling Velcro fasteners, tying shoelaces, using clips, fitting pieces together, opening and closing taps, turning door handles and locks, as well as opening and closing cream jars. To increase the complexity of the exercise, the use of materials of different sizes is recommended, preferably of progressively smaller dimensions. Figure 7 illustrates the exercise described.

#### 3.3.5. Evaluation of Outcomes

Monitoring during the performance of the Nine Hole Peg Test makes it possible to assess progress in carrying out movements that require fine motor function. At the same time, observation of the person while performing activities of daily living, as well as the person’s own perception, allow the evaluation of the gains achieved. If the interventions implemented result in improvements, the following are highlighted as positive outcomes: (1) Improved fine motor function; (2) Improved awareness of the relationship between exercises and fine motor function; (3) Improved knowledge about fine motor function exercises; (4) Improved ability to perform fine motor function exercises; (5) Improved meaning attributed to fine motor function training. With continued positive progression, the judgement “improved” may evolve and reach the judgement “effective”.

## 4. Discussion

The present study made it possible to develop and validate, based on expert consensus, the design of rehabilitation nursing care directed towards older adults with impaired fine motor function. The proposed model advances rehabilitation nursing by systematising fine motor rehabilitation within a structured clinical framework. Compared with existing international models, our design emphasises the unique nursing contribution to education, engagement, functional adaptation and self-care. The structuring into five steps, aligned with the nursing process (data collection, diagnoses, objectives, planning of interventions, and evaluation), highlights the relevance of a systematised model that can guide clinical practice, ensuring an individualised, intentional, and scientifically grounded approach [40].

The use of the e-Delphi technique proved to be appropriate, as it enabled the integration of the specialised knowledge of expert nurses in rehabilitation nursing, ensuring the robustness of the content and its practical applicability. The high level of consensus achieved (CVI ≥ 0.90 in all steps) confirms the relevance of the content included and demonstrates that the model can constitute a reliable guiding framework for interventions with this population. This aspect is particularly relevant given the scarcity of structured references regarding specific interventions for fine motor function in the field of rehabilitation nursing.

In addition to the robustness and confirmed practical applicability, an innovative contribution lies in the emphasis on the adaptive process of the older person by integrating diagnoses related to awareness, knowledge, and the meaning attributed to fine motor function training, in line with several authors [41]. This focus allows fine motor function to be understood not only as a physical dimension but also as a multifaceted experience involving perception, motivation, and psychosocial adaptation.

Despite the relevance attributed to fine motor function, it is known that this domain tends to be frequently relegated to a secondary level in care planning, particularly in the early stages of hospitalisation [30]. The model developed in this study helps to counter this trend by proposing the systematisation of intervention in this domain, thereby facilitating and guiding the practice of the SNRN.

The systematisation of specific fine motor function exercises and the inclusion of diversified resources graded in terms of complexity are in line with the recommendations of recent studies [12,23,25], which highlight the importance of therapeutic progression and the adaptation of exercises to the person’s individual capacities. Indeed, the use of exercises graded in terms of complexity promotes not only functional recovery but also the person’s motivation and adherence to the rehabilitation process.

In this context, Pérez-Mármol et al. (2017) investigated the effectiveness of a rehabilitation programme focused on hand fine motor function [42]. The interventions were carried out three times per week, for 45 min, over eight weeks. Participants were instructed to make small paper balls using circular opposition movements between the thumb and index finger. The intensity of the programme was progressively increased by varying the size and number of balls produced. The results showed that the intervention contributed to improvements in manual dexterity and finger range of motion, aspects that are fundamental for daily functionality and for enhancing quality of life.

Similarly, Maio et al. (2022) highlighted the benefits of a structured programme of diversified exercises to improve manual dexterity and motor coordination [43]. The protocol included tasks such as folding pieces of paper into different shapes (3 sets of 5 repetitions), touching a musical mat with the fingers (3 min), creating figures with paper (5 min), finger painting (5 min), tracing drawings (3 min), making bracelets (5 min), counting coins and placing them in a money box (5 min), opening and closing a bottle (3 sets of 10 repetitions), stamping on paper (3 sets of 10 repetitions), modelling letters with modelling clay (3 min), and manipulating blocks on the modelling clay (3 min). The sessions, conducted twice a week, lasting 60 min over 12 weeks, resulted in significant improvements in fine motor function, leading to greater independence in performing activities of daily living such as dressing, eating, and carrying out tasks requiring manual coordination.

In line with these results, the rehabilitation nursing care design model for older adults with impaired fine motor function developed in this study stands out by emphasising self-care activities within the exercise plan, in alignment with the regulatory instruments of the professional practice of SNRN [29,31]. This integration underscores the need to promote healthier developmental and health–disease transitions, ensuring that the intervention is not limited to motor training but enhances effective and meaningful functional gains in the daily lives of older adults.

Fine motor function is intrinsically linked to the person’s motivation and the demands of their daily life, playing a decisive role in the performance of activities of daily living as well as in professional and leisure tasks. The true purpose of rehabilitation lies in the ability to integrate these activities into the person’s routine, thereby promoting independence and, consequently, quality of life [25,28]. In this sense, the care design model developed incorporated this perspective by structuring exercises that reproduce everyday activities, with particular emphasis on those related to self-care, ensuring their practical and functional relevance.

In the context of functional hand rehabilitation, it is essential to associate the development of motor skills with the psychosocial framework, ensuring that the rehabilitation process is both motivating and meaningful. Adherence to the rehabilitation plan largely depends on the person’s active involvement and on the value they attribute to the proposed interventions [28,43]. In this regard, the care design model developed in this study also stands out, as it emphasises the adaptation of interventions to the older person’s life context and prioritises strategies that promote awareness and engagement in the rehabilitation process.

The implementation of fine motor function exercises should include the use of easily handled objects, such as cubes or cones, which encourage basic pincer and grasping movements. In addition, the introduction of materials such as modelling clay, elastic bands, or pegs allows variation in resistance and increases task complexity, facilitating gradual adaptation to more demanding challenges. The incorporation of familiar tools and activities also represents a valuable strategy, as it enhances motivation and the perceived usefulness of the tasks performed. In the proposal developed, these principles were explicitly integrated, prioritising activities that stimulate the repetition of movements, proprioception, resistance, and muscle strength. Within this framework, the diversification of materials and exercises was adopted as a structuring resource, enabling progressive adjustment to motor demands and contributing to the optimisation of functional recovery [24,28].

Additionally, it is important to highlight the incorporation of the Nine Hole Peg Test as an assessment tool [12,23] and as support for clinical decision-making in rehabilitation nursing, centred on measurable outcomes and oriented towards health gains. The use of this tool provides objectivity in monitoring the progression of fine motor function and facilitates critical analysis of the impact of interventions, enabling evidence-based decision-making and dynamic adjustment of the care plan.

The final model of the rehabilitation nursing care design directed towards older adults with impaired fine motor function, enriched with photographic illustrations of some exercises, increases its potential for practical application by health professionals. This aspect also favours its future transferability to other contexts—such as long-term care units, day centres, or even the home—thereby facilitating continuity of care and improvement of fine motor function after hospital discharge [44].

In addition to content validation, this study makes a pioneering contribution to the systematisation of the role of the SNRN in the care of older adults with impaired fine motor function. Although guiding documents from the Portuguese Nursing Council recognise the specificity of this role [29,31], until now there has been a lack of a structured and validated tool. The proposal presented emerges as a concrete response to this gap, offering a practical, evidence-based instrument validated by experts, with the potential to draw the attention of nursing professionals to the need to rethink current practices and to improve the quality of care provided [45].

When positioned within the landscape of international rehabilitation frameworks, the proposed model adds a unique nursing-centred contribution. While the ICF provides a global conceptual structure for understanding functioning and disability, and occupational therapy–driven models (such as the Model of Human Occupation or the Canadian Model of Occupational Performance) offer valuable perspectives on activity and participation, none provide operational guidance tailored to nursing practice—particularly regarding systematic assessment, diagnosis, planning and evaluation of fine motor rehabilitation in older adults. The present model fills this gap by translating international concepts into a structured, clinically applicable framework aligned with the SNRN scope of practice, thereby enhancing the specificity and usability of rehabilitation guidance within nursing contexts.

### 4.1. Study Limitations

Despite its contribution, this study presents some limitations that should be acknowledged. The first concerns the fact that validation was carried out exclusively with experts from the national context, which may limit the international generalisability of the results. Inclusion of professionals from other rehabilitation disciplines could enrich future validations.

Furthermore, the use of purposive sampling, although appropriate in the context of an e-Delphi, may introduce some selection bias, since the experts selected share similar professional trajectories, which may have influenced the diversity of perspectives.

A second limitation relates to the absence of empirical application of the model with the target population, which currently prevents confirmation of its clinical effectiveness.

### 4.2. Implications for Clinical Practice

The implementation of this care design model has significant implications for clinical practice, by providing a structured framework that contributes to the standardisation of the SNRN’s intervention in the domain of fine motor function. By systematising care, the model promotes greater consistency in practices across different contexts of practice, reinforcing the safety and quality of the intervention. At the same time, by integrating exercises focused on self-care, it establishes a direct link between motor training and functionality, thereby enhancing effective gains in terms of autonomy, social participation, and quality of life of the older person.

It should also be highlighted that this model values the specific role of the SNRN, by providing concrete tools to support clinical decision-making and to document the outcomes achieved.

From a practical standpoint, adoption of this model may standardise SNRN interventions, reduce variability in clinical practice and support more precise monitoring of functional outcomes. Its structure, anchored in the nursing process, may also strengthen interdisciplinary communication by clarifying the specific contribution of rehabilitation nursing to fine motor recovery. Educationally, the model provides a pedagogical resource that can be integrated into postgraduate rehabilitation nursing curricula, enabling students and specialists to develop competencies in systematic fine motor assessment and intervention. At an organisational level, the model may support the development of clinical protocols, documentation standards and quality indicators, contributing to more efficient care pathways and improved continuity of care across settings, including acute care, inpatient rehabilitation, long-term care and home-based services.

### 4.3. Implications for Future Research

In view of these limitations, it is recommended that longitudinal and multicentre studies be conducted to test the effectiveness of the care design model in different practice settings and with diverse populations, in order to benefit an increasing number of older adults undergoing rehabilitation. It would also be relevant to explore the perceptions of older adults themselves and their caregivers regarding the applicability and impact of the exercises described, thereby promoting the inclusion of multiple perspectives in the design of rehabilitation nursing care.

Another relevant line of research consists of assessing the integration of the model into digital or telehealth rehabilitation programmes, considering the growing investment in technologies to support rehabilitation (e.g., online platforms, mobile applications, virtual reality).

Future research should focus on empirically testing the clinical effectiveness of the model through longitudinal or multicentre studies, examining its impact on functional outcomes, self-care performance and quality of life. Implementation studies could explore the feasibility of integrating the model into digital rehabilitation platforms or tele-rehabilitation programmes, given the growing use of technology to support remote therapeutic engagement. Further investigation is also warranted to identify organisational facilitators and barriers to the adoption of the model, as well as to evaluate its applicability in international contexts, which may contribute to refining the framework and enhancing its global relevance.

## 5. Conclusions

The demographic shift, marked by the progressive ageing of the population and the increasing prevalence of chronic diseases, reinforces the need to equip health professionals with appropriate knowledge to promote and restore fine motor function, given its direct impact on the autonomy and quality of life of older adults. This domain is particularly relevant as it cuts across several areas of competence of the SNRN, namely neurological, cardiorespiratory, and musculoskeletal.

An intervention aimed at the recovery of fine motor function proves to be crucial, particularly in activities related to self-care, as it facilitates the adaptive process by supporting the person’s readjustment to the limitations imposed by ageing and disease. Rehabilitation focused on this function, through the development of specific exercises, has the enhancement of independence and the promotion of better quality of life as its ultimate goals. In this sense, investment in fine motor function plays a central role in the person’s overall recovery, not only by fostering autonomy but also by contributing to the reduction in the perception of disability and to the improvement of social participation.

In line with the above, the present study enabled the development and validation, based on expert consensus, of the design of rehabilitation nursing care directed towards older adults with impaired fine motor function. The structuring of the model into five steps, aligned with the nursing process, highlights the relevance of a systematised framework that supports clinical practice, ensuring an individualised, intentional, and evidence-based approach.

By organising assessment, diagnosis, intervention planning and evaluation in a coherent sequence, the model addresses a recognised gap in rehabilitation nursing: the lack of structured and operational guidance specifically focused on fine motor rehabilitation in older adults. This contributes to greater clarity in the role of the SNRN and may help standardise practice across different care settings.

Looking ahead, further research is needed to explore the model’s applicability and effectiveness in clinical practice. Pilot studies or clinical trials could examine its impact on functional outcomes, self-care performance and quality of life, while implementation studies may identify facilitators and barriers to its integration into routine care. The model could also be adapted for use in different health systems or incorporated into digital or home-based rehabilitation programmes. These developments would strengthen its utility and support its evolution as a practical resource for rehabilitation nursing.

In summary, this study provides a structured and validated contribution to the systematisation of rehabilitation nursing care, offering a model that can support clinical decision-making, promote health gains and reinforce the role of SNRNs in the recovery of older adults with impaired fine motor function.

## Figures and Tables

**Table 1:** Sociodemographic and professional characterisation of the experts.

Sociodemographic and Professional Characteristics	First and Second Rounds (n = 15)
Gender n (%)	
Female	9 (60.0%)
Male	6 (40.0%)
Age (years) Mean; Std. Dev.	38.3; ±8.2
Education n (%)	
Bachelor’s degree	7 (46.7%)
Master’s degree	8 (53.3%)
Job Title n (%)	
Nurse with certified advanced training in rehabilitation nursing	5 (33.3%)
Nurse Specialist	10 (66.7%)
Area of specialisation in Nursing n (%)	
Rehabilitation nursing	15 (100%)
Time of professional practice (years) Mean; Std. Dev.	16.3; ±8.2

**Table 2:** Validation of the content of the proposed care design.

Steps of the Care Design	CVI First Round	CVI Second Round
Collection of relevant data	1.00	1.00
Identification of possible nursing diagnoses	0.89	1.00
Definition of objectives	1.00	1.00
Planning and implementation of interventions	0.89	1.00
Evaluation of outcomes	1.00	1.00

CVI—Content Validity Index.

## Data Availability

The data that support the findings of this study are available from the corresponding author upon reasonable request. The data are not publicly available due to privacy and ethical restrictions.

## Public Involvement Statement

No public involvement in any aspect of this research.

## Guidelines and Standards Statement

This manuscript was drafted against the CREDES (Conducting and Reporting Delphi Studies) guidance for Delphi study research.

## Abbreviations

The following abbreviations are used in this manuscript:

ADL	Activities of Daily Living
CVI	Content Validity Index
ICF	Classification of Functioning, Disability and Health
ICNP	International Classification for Nursing Practice
SNRN	Specialist Nurses in Rehabilitation Nursing
WHO	World Health Organization
